# Diagnostic accuracy of computed tomography angiography (CTA) for diagnosing blunt cerebrovascular injury in trauma patients: a systematic review and meta-analysis

**DOI:** 10.1007/s00330-021-08379-7

**Published:** 2021-12-02

**Authors:** Charlotte C. Kik, Willem-Bart M. Slooff, Nizar Moayeri, Pim A. de Jong, Sander P. J. Muijs, F. Cumhur Öner

**Affiliations:** 1 Department of Neurosurgery, University Medical Center Utrecht (UMCU), Heidelberglaan 100, 3584DX Utrecht, The Netherlands; 2 Department of Radiology, University Medical Center Utrecht (UMCU), Utrecht, The Netherlands; 3 Department of Orthopaedic Surgery, University Medical Center Utrecht (UMCU), Utrecht, The Netherlands

**Keywords:** Cerebrovascular trauma, Wounds, nonpenetrating, Angiography, digital subtraction, Computed tomography angiography

## Abstract

**Objectives:**

Previous literature showed that the diagnostic accuracy of computed tomographic angiography (CTA) is not equally comparable with that of the rarely used golden standard of digital subtraction angiography (DSA) for detecting blunt cerebrovascular injuries (BCVI) in trauma patients. However, advances in CTA technology may prove CTA to become equally accurate. This study investigated the diagnostic accuracy of CTA in detecting BCVI in comparison with DSA in trauma patients.

**Methods:**

An electronic database search was performed in PubMed, EMBASE, and Cochrane Library. Summary estimates of sensitivity, specificity, positive and negative likelihood, diagnostic odds ratio, and 95% confidence intervals were determined using a bivariate random-effects model.

**Results:**

Of the 3293 studies identified, 9 met the inclusion criteria. Pooled sensitivity was 64% (95% CI, 53–74%) and specificity 95% (95% CI, 87–99%) The estimated positive likelihood ratio was 11.8 (95%, 5.6–24.9), with a negative likelihood ratio of 0.38 (95%, 0.30–0.49) and a diagnostic odds ratio of 31 (95%, 17–56).

**Conclusion:**

CTA has reasonable specificity but low sensitivity when compared to DSA in diagnosing any BCVI. An increase in channels to 64 slices did not yield better sensitivity. There is a risk for underdiagnosis of BCVI when only using DSA to confirm CTA-positive cases, especially in those patients with low-grade injuries.

**Key Points:**

• *Low sensitivity and high specificity were seen in identifying BCVI with CTA as compared to DSA.*

• *Increased CTA detector channels (≤ 64) did not lead to higher sensitivity when detecting BCVI.*

• *The use of CTA instead of DSA may lead to underdiagnosis and, consequently, undertreatment of BCVI.*

## Introduction

Blunt cerebrovascular injuries (BCVI) collectively describe all non-penetrating traumatic injuries to the extra- or intracranial carotid and vertebral arteries. The mechanism of injury is either high-energy flexion, extension, or rotation of the neck, or a direct blow to or laceration of the blood vessels. At the level of the vessel wall, there is a risk of tear formation of the tunica intima because of the increased arterial strain. Expedited by a trauma-induced state of hypercoagulability caused by the initial trauma, the exposed subendothelial collagen activates the coagulation cascade, leading to an intraluminal thrombus formation at the site of the tear or a complete vessel occlusion. The arterial defect can also be a gateway for blood to enter the underlying layers of the vessel wall and can cause the formation of a traumatic (pseudo) aneurysm. As a result, patients with a BCVI are at risk of a secondary brain injury caused either by thromboembolism or occlusion of the artery [1–4].

Previously, BCVI was considered a rare cause of cerebral ischemia and ischemic stroke. Due to improved diagnostic imaging modalities, awareness, and the introduction of standard screening protocols, such as the Memphis and (modified) Denver criteria, the reported incidence of BCVI among blunt trauma patients has increased over recent years [5–9]. The prevalence ranges from 1–2% in patients with blunt trauma to 9% in patients with a severe head injury [3, 10, 11]. When comparing BCVI to non-traumatic brain injuries such as stroke, BCVI is associated with poorer cognitive outcomes, although long-term outcomes following BCVI are missing [12].

There is a 72-h window after injury to provide anti-aggregation and anticoagulation therapy to reduce the risk of secondary brain injury [4]. Screening of patients suspected of BCVI remains pivotal as up to 80% of these patients do not display neurological symptoms at presentation [13, 14]. The golden standard for diagnosing BCVI is digital subtraction angiography (DSA). However, non-invasive and fast screening modalities such as computed tomography angiography (CTA) are increasingly utilized in the acute phase [15–18]. A previously published meta-analysis showed great variability in the sensitivity of BCVI detection using CTA when compared to DSA [19]. Although DSA is the golden standard to date, recent data suggest that CTA with 64 channels has comparable sensitivity rates in diagnosing BCVI and could potentially replace DSA [19, 20]. Therefore, we performed a systematic review and meta-analysis to evaluate the contribution of new data on CTA sensitivity in diagnosing BCVI [19]. We hypothesized that CTA would result in similar accuracy for diagnosing BCVI compared to the golden standard DSA.

## Material and methods

### Literature search

Studies containing CTA as diagnostic imaging for BCVI that were published until February 24, 2021, were screened independently by two investigators (C.C.K., W.B.S.). An electronic database search was performed in both PubMed, EMBASE, and Cochrane Library using the following keywords: carotid, carotid artery, vertebral artery, intracranial, extracranial, neck, vertebral and vascular system, combined with blunt wound, or blunt trauma, or nonpenetrating injury/wound using the Boolean operator AND for the population. The index and reference test were defined using the keywords: computed tomography angiography, CTA, angiography, and angiotomography, digital subtraction angiography, digital subtraction arteriography, DSA, cerebral angiography, and diagnosis. Additional publications were identified through citation chaining of the bibliography of reviews and other potentially relevant studies.

### Study selection and data extraction

Both investigators (C.C.K., W.B.S.) reviewed titles and abstracts for relevance and identified potentially relevant citations for full-text review using the online reviewing tool Rayyan (http://rayyan.qcri.org) [21]. The complete inclusion and exclusion criteria are shown in Table 1.

**Table 1:** Inclusion and exclusion criteria

Inclusion criteria	Exclusion criteria
*Population*: Age ≥ 16 with blunt trauma suspected of BCVI identified by the Denver or modified Memphis criteria. *Intervention:* multidetector CT angiography *Study design*: primary studies on diagnostic accuracy *Data:* presented allowing two-by-two contingency table construction.	Case reports, editorials, and opinions Case studies with less than five patients Unoriginal and unpublished studies. Studies with missing data / full text irretrievable: In case of protocol and publication: at least 1 try for contact to gain the article. In case the article was not available: at least 1 try with the department's secretary for gaining the article.

Investigators extracted the subsequent data: study design; date of patient screening; study location; inclusion and exclusion criteria; number of patients included; number of patients excluded; number of patients included in the final analysis; reference and index test; mean age and gender of participants; primary unit of analysis; who reviewed the reference and index test and whether this was done blinded; the arteries examined for BCVI; the type of CT scanner and DSA equipment; the number of slices; slice thickness; size interval; level of reconstruction; injection rate; type of contrast used in CTA and DSA; typical contrast volume used in CTA and DSA; true positives, negatives, false positives, and negatives.

### Assessment of methodological quality

Methodological quality was assessed using the Quality Assessment of Diagnostic Accuracy Studies, second version (QUADAS-2), which investigates both risks of bias as well as applicability concerns [22]. QUADAS-2 uses 7 questions to assess study selection and setting, conduct, and interpretation of the reference and index test, and flow and timing using three levels of bias (high, low, and unclear). Patient selection was considered at low risk of bias when a consecutive or random sample of patients was enrolled, when the case-control design was avoided, and when a study avoided inappropriate exclusions. A low-risk setting was one in which all patients underwent both CTA and DSA. Additionally, there was a low risk of bias in the interpretation of the index and/or reference test if the reviewers had no prior knowledge of the results of the reference test or the index test. A maximum of 48-h interval between CTA and DSA is presumed to be appropriate [19].

### Data synthesis

Some studies reported data on blunt carotid artery injury (BCVI_*carotid*_) or blunt vertebral artery injury (BCVI_*vertebral*_) separately but did not include data on BCVI per patient [6, 19, 23–25]. Patients diagnosed with BCVI could potentially have multiple injuries to either or both the carotid and vertebral arteries. Therefore, studies that only reported results for BCVI_*carotid*_ and BCVI_*vertebral*_ did not allow for calculation of diagnostic accuracy per patient. Instead, all data were combined in one larger overall group called “any BCVI.” In this group, true and false positives and negatives were either assessed per patient and/or from the combined results of BCVI_*carotid*_ and BCVI_*vertebral*_ if no per-patient data was given. Separate analyses on diagnostic accuracy were also performed for BCVI_*carotid*_ and BCVI_*vertebral*_ .

When true and false positive and negative findings were separately reported by different radiologists, the average of each observation was calculated for that study population. Additionally, and if reported separately, the sum of the common, cervical, or intracranial carotid artery was calculated to determine BCVI_*carotid*_. Likewise, the sum of the cervical or intradural vertebral artery was calculated to determine BCVI_*vertebral*_.

### Statistical analysis

True and false positives and negatives were used to individually calculate the sensitivity and specificity of CTA for each study. The 95% confidence interval was calculated using the Clopper-Pearson interval method [26]. Summary estimates of sensitivity, specificity, positive and negative likelihood, and diagnostic odds ratio and their 95% confidence intervals were determined for CTA in BCVI_*carotid*_, BCVI_*vertebral*_, and any BCVI using a bivariate random-effects model. This allowed the heterogeneity beyond chance between studies to be considered. The percentage of total variation across studies was evaluated using forest plots, chi-square, Cochrane Q, and *I*^2^, which measures the impact of unobserved heterogeneity [27]. We considered *I*^2^ values between 0 and 50% as medium heterogeneity, while values > 50% were considered high heterogeneity, and *p* values < 0.05 significant.

Hierarchical summary receiver operating characteristic (SROC) plots were created for visual assessment of the threshold effect by calculating the squared correlation coefficient estimate from the between-study covariance parameter [28]. Within the SROC plot, observed data points, a summary operating point of sensitivity and specificity, and 95% confidence interval contour were displayed.

Cook’s distance was determined to analyze the influence of each study [29]. Outliers were evaluated using scatter plots using standardized predicted random effects and bivariate box plots [30]. Publication bias was assessed using Deek’s funnel plot asymmetry (*p <* 0.10 indicating significant asymmetry) [31]. To explore sources of heterogeneity, subgroup analyses, and univariate meta-regression was used. All analyses, except subgroup analyses and univariate meta-regression, were performed using STATA 16.0 (StataCorp. 2019. *Statistical Software*: *Release 16*: StataCorp LLC.) in combination with the MIDAS command [32]. Subgroup analyses and meta-regression were performed using Open Meta Analyst [33].

## Results

### Study selection and inclusion

**Fig. 1:**
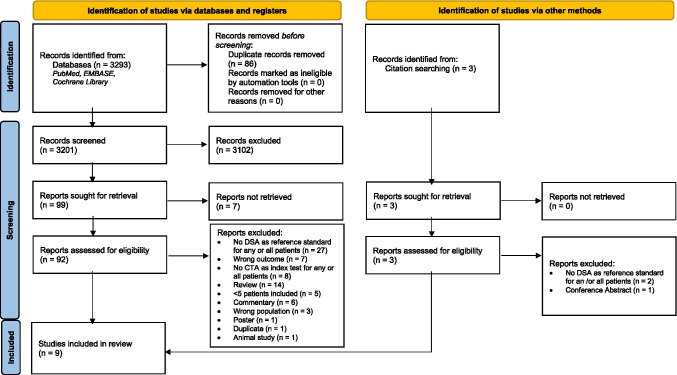
PRISMA Flow Diagram for study selection and inclusion

Our electronic database search of PubMed, EMBASE, and the Cochrane Library yielded 3293 studies, of which 92 studies were identified as duplicates. Of the 3204 studies screened for title and abstract, 102 articles were found eligible for full-text assessment. subsequently, 93 articles were excluded for various reasons, including not having DSA as a reference test for any and/or all patients (29 studies), not reporting the outcome or population of interest (21 studies), no CTA as an index test for any and or all patients (8 studies), non-original studies (14 review studies), wrong study design (14 studies), and no full-text availability (7 studies). Finally, 9 studies were included for quality assessment (Fig. 1) [6, 20, 23–25, 34–37].

### Quality assessment

**Fig. 2:**
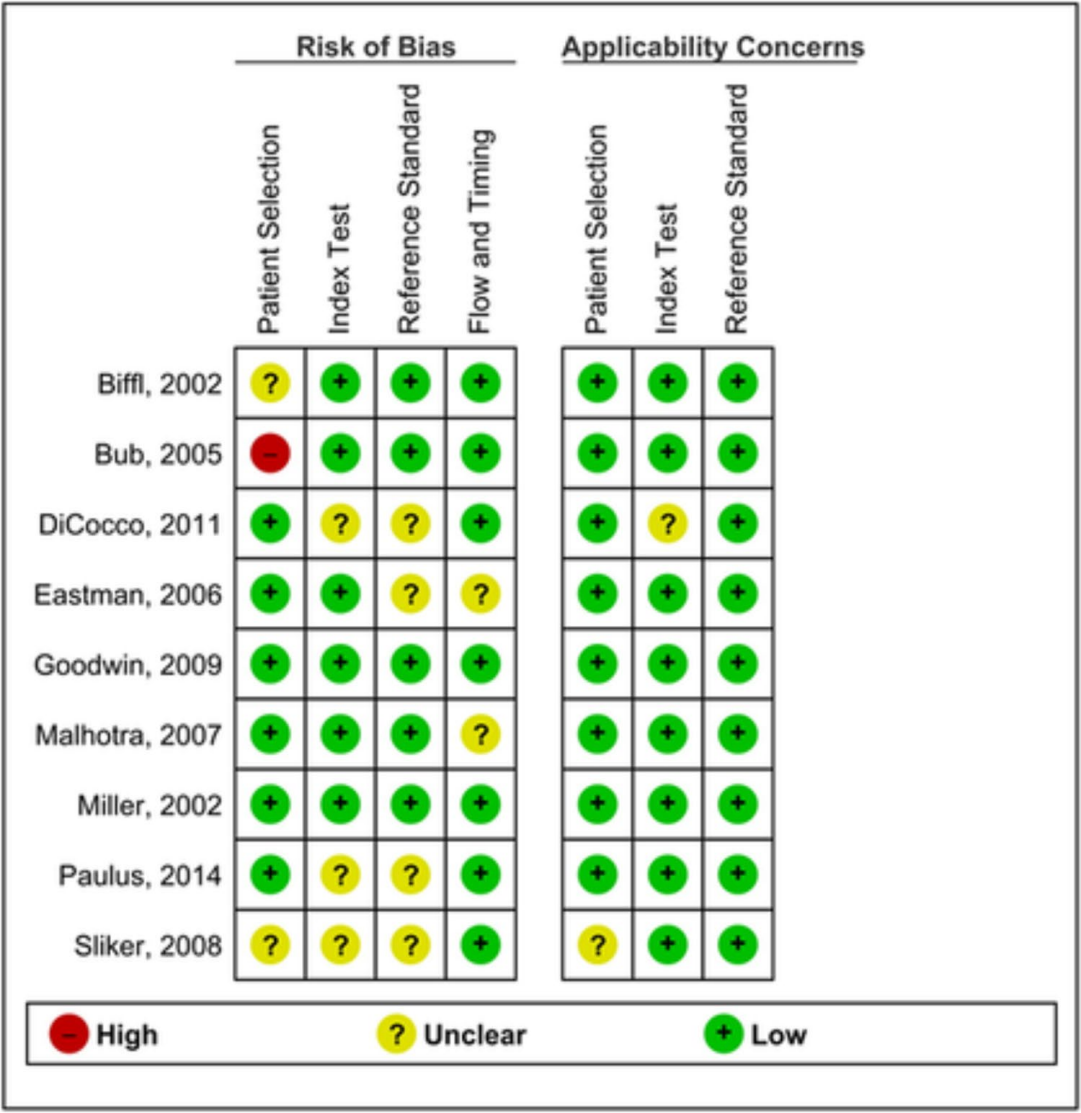
QUADAS-2 risk of bias assessment and applicability concerns

The risk of bias and applicability were assessed using QUADAS-2 tool questions for all included (Fig. 2). Two studies reported no or unclear data on whether patients were consecutively enrolled or randomly selected, or whether a case-control design was avoided [25, 34]. One study systematically applied CTA in patients suspected of BCVI and used DSA in a minority of those patients [23]. They, therefore, anticipated a higher rate of positives in their study population. We also anticipated high risk of bias in patient selection for this study. Three studies clearly stated whether the results of the index test were interpreted without prior knowledge of the results of the reference test [20, 24, 25]. This was also the case for four studies regarding the reference test [20, 24, 25, 35]. In two studies, it was unclear whether all patients were included in the analysis [35, 37]. Overall, there were no concerns for applicability with either of the studies included. The risk of bias was not considered great enough to exclude any study from further analysis.

### Study characteristics

All study and patient characteristics are summarized in Table 2. Of the 9 included studies, 3 were conducted retrospectively, 5 prospectively, and one both retrospectively and prospectively. A total of 1918 patients were screened for BCVI with both DSA and CTA, 67% male with an overall mean age of 40.5 years (range 1–94).

**Table 2:** Study and patient characteristics of the studies included

		Study period						Gender			
Study	Location	From	Until	Temporality	Index test (slices)	Reference test	No. patients screened with CTA and DSA	Male	Female	Mean age (years)	Scans reviewed by
Paulus et al [20]	Memphis, Tennessee, USA	May 2011	May 2012	Retrospective	CTA (64)	DSA	594	373	221	43	Attending neuroradiologist
Dicocco et al [24]	Memphis, Tennessee, USA	January 2007	May 2009	Retrospective	CTA (32)	DSA	684	474	210	39	NA
Goodwin et al [36]	Columbus, Ohio, USA	June 2007	February 2008	Prospective	Cervical CTA (16)	DSA	158	117	41	42	(interventional) Radiologist
Sliker et al [25]	Baltimore, Maryland, USA	May 2004	November 2004	Mixed	Cervical CTA (16 and 64)	DSA	48	NA	NA	NA	Trauma radiologist
Malhotra et al [37]	Richmond, Virginia, USA	December 2003	March 2007	Prospective	Cervical CTA (16)	DSA	89	54	40	38	Attending radiologist and senior radiology resident
Eastman et al [35]	Dallas, Texas, USA	April 2004	February 2005	Prospective	Helical CTA (16)	DSA	124	NA	NA	NA	Attending neuroradiologist
Bub et al [23]	Seattle, Washington, USA	January 2001	March 2002	Retrospective	Cervical CTA (1, 4, and8)	DSA	32	NA	NA	NA	Two neuroradiologists, one third-year radiology resident.
Miller et al [6]	Memphis, Tennessee, USA	January 2000	March 2002	Prospective	Helical CTA (1)	DSA	143	NA	NA	37.6	Staff neuroradiologists
Biffl et al [34]	Denver, Colorado, USA	April 1996	June 2001	Prospective	Helical CTA (4)	DSA	46	NA	NA	NA	Radiologists experienced in neurovascular imaging

Six studies used CTA with 16 slices or more [20, 24, 25, 35–37]. One study reported both data for 16 and 64 slices CTA [36]. We chose to consider these as two separate data sets in our analyses. Most studies used CTA with Omnipaque (Amersham Health Inc.), with varying concentrations between 300 and 350 mg/mL [35–37]. DSA was used as a reference test in all studies, with descriptions on model and contrast type being noted in almost all except for two studies [6, 34]. A complete summary of the geographical information for all commercial products is shown in Appendix 1.

All diagnostic accuracy estimates are listed in Table 3. Overall, three studies reported both outcomes for BCVI per-patient and per-artery [20, 35, 37]. The per-patient data were used to calculate true and false positives and negatives in the category “any BCVI.” If unavailable, the sum of both carotid and vertebral injuries was used.

**Table 3:** Pooled sensitivity, specificity, positive and negative likelihood ratio’s, diagnostic odds ratio’s and funnel plot’s asymmetry for CTA vs DSA per category

		Mean value (95% CI)					
Category	No. of studies	Sensitivity (%)	Specificity (%)	Positive LRϕ (%)	Negative LRϕ (%)	DOR*	Funnel plot asymmetry (*p* value)
Any BCI [6, 20, 23–25, 34, 36, 37]	8	64 [0.53–0.74]	0.95 [0.87–0.98]	11.8 [5.6–24.9]	0.38 [0.30–0.49]	31 [17–56]	0.914
BCVI_carotid_ [6, 20, 23–25, 35, 37]	7	70 [52–84]	98 [94–99]	35.4 [10.3–121.3]	0.31 [0.17–0.53]	116 [23–583]	0.187
BCVI_vertebral_ [6, 20, 23–25, 35, 37]	7	70 [55–82]	99 [0.94 –1.00]	47.8 [10.3–221.8]	0.30 [0.19–0.48]	158 [26–962]	0.474

*Diagnostic odds ratio, ϕLikelihood ratio, *BCVI* blunt cerebrovascular injury

### Outlier detection, influence analysis, and publication bias

Using standardized predicted random effects and bivariate box plots, outliers were identified for all four categories (per-patient, BCVI_*carotid*_, BCVI_*vertebral*_, and any BCVI). In one study, outliers were detected in the category “any BCVI” [35]. Additionally, Cook’s distance depicted the same study as an outlier.

Publication bias was assessed using Deek’s funnel plot asymmetry test and visual funnel plot analysis. There was no significant asymmetry between studies in the four different categories, with *p* values ranging between 0.187 for BCVI_*carotid*_ and 0.914 for any BCVI (Table 6). No concern for publication bias was found.

### Pooled diagnostic accuracy estimates for CTA versus DSA for any form of BCVI

**Fig. 3:**
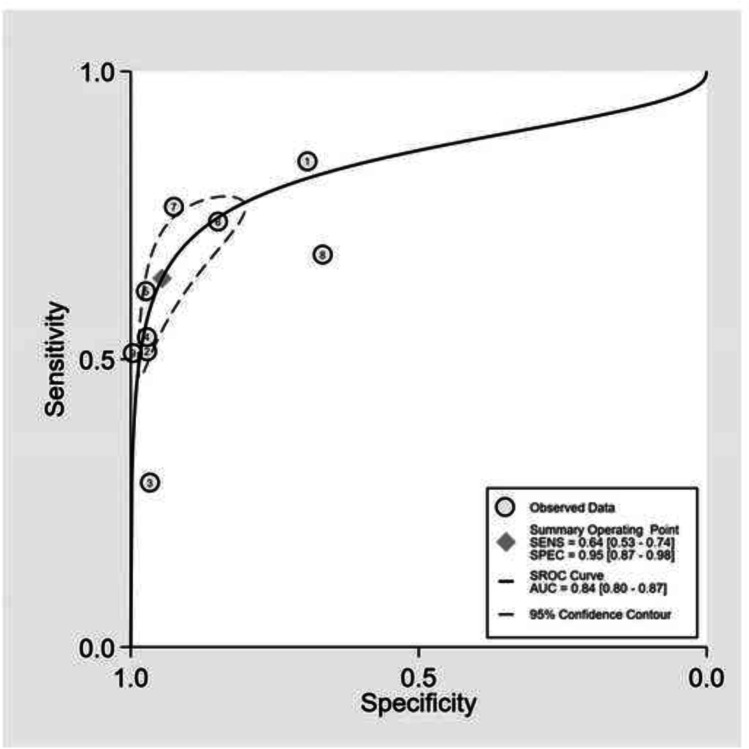
Summary Operating Characteristic (SROC) plot for sensitivity and specificity of CTA vs DSA in diagnosing BCVI

**Fig. 4:**
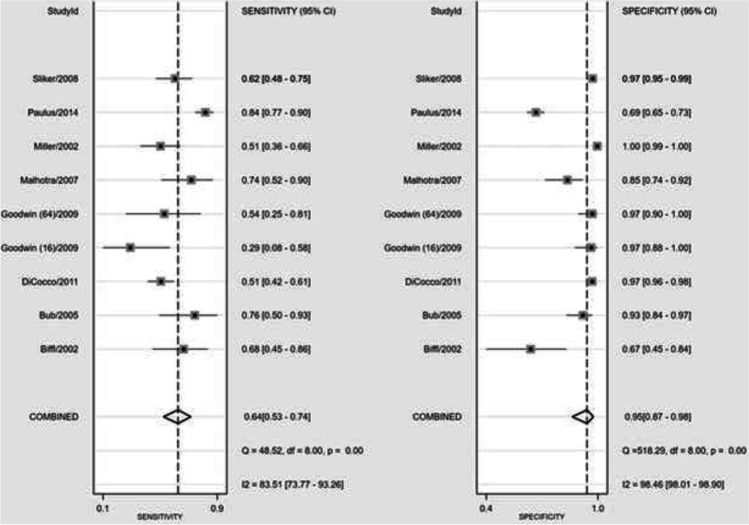
Forest plot for pooled sensitivity and specificity for CTA vs DSA in diagnosing BCVI

**Fig. 5:**
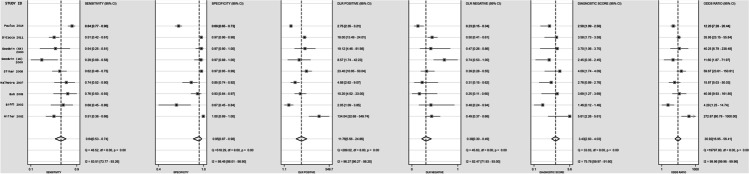
Forest plot for pooled sensitivity, specificity, positive and negative likelihood ratios, diagnostic scores and diagnostic odds ratios for CTA vs DSA in diagnosing BCVI

After outlier removal, 8 studies were included in the analysis for diagnostic accuracy estimates of CTA versus DSA for any BCVI [6, 20, 23–25, 34, 36, 37]. Figure 3 shows the SROC plot for the detection of any BCVI with CTA vs DSA and the covariation in sensitivity and specificity of the studies included. Pooled sensitivity was 64% (95% CI, 53–74%) and specificity 95% (95% CI, 87–99%) (Figs. 4 and 5). There was a high degree of heterogeneity between studies, with an *I*^2^ for sensitivity of 76.07 (*p* < 0.01*)* and specificity of 95.20 (*p* < 0.01) (Fig. 4). Forest plots for the pooled diagnostic odds ratio and positive and negative likelihood ratio are provided in Appendix 2.

## Pooled diagnostic accuracy estimates for CTA versus DSA in BCVI_*carotid*_ and BCVI_*vertebral*_

To determine diagnostic accuracy for CTA vs DSA in BCVI_*carotid*_ and BCVI_*vertebral*_, data were combined from 7 studies [6, 20, 23–25, 35, 37]. Pooled sensitivity was 70% (95% CI, 52–84%) and specificity 98% (95% CI, 94–99%) in BCVI_*carotid*_ (Table 3). Pooled sensitivity was 70% (95% CI, 55–82%) and specificity 99% (95% CI, 94–100%) in BCVI_*vertebral*_ (Table 3).

There was a high degree of heterogeneity between studies, with an *I*^2^ for sensitivity of 86.87 (*p* < 0.01) and specificity of 97.64 (*p* < 0.01) for BCVI_*carotid*_ and an *I*^2^ for sensitivity of 76.07 (*p* < 0.01) and specificity of 95.20 (*p* < 0.01) in BCVI_*vertebral*_.

### Subgroup analyses and meta-regression

Exploration of the sources of heterogeneity found there was no significant difference between studies based on publication year and the number of CT detector rows when calculating combined pooled sensitivity and specificity of CTA for the detection of any BCVI (Table 4). There was a significant difference, however, when comparing per-artery and per-patient studies. Sensitivity was reported at 70.3% (95% CI, 41.3–88.9) in ≥ 16-slice CTA vs 63.1% (95% CI, 46.3–77.2) in < 16-slice CTA (*p *= 0.868), and specificity at 96.1 (95% CI, 86.5–98.9) in ≥ 16-slice CTA vs 94.6 (95% CI, 61.–99.5) in < 16-slice CTA (*p *= 0.683).

**Table 4:** The exploration of heterogeneity via subgroup analysis and meta-regression by identifying covariates in the estimated combined pooled sensitivity and specificity of CTA for detection of any BCVI

	No.		Mean value (95% CI)		
Covariate	Studies	Patients	Pooled sensitivity %	Pooled specificity %	Meta-regression joint *p*
Temporality					
Prospective [6, 34–37]	5	610	64.9 [44.7–80.8]	96.0 [84.5–99.1]	
Retrospective [20, 23–25]	4	1363	69.5 [49.2–84.2]	93.1 [69.7–98.8]	0.029
Number of CTA slices					
<16 [6, 23, 34]	3	226	63.1 [46.3–77.2]	94.6 [61.7–99.5]	
16 [25, 35–37]	4	385	70.3 [41.3–88.9]	96.1 [86.5–98.9]	0.868*
>16 [20, 24, 36]	3	1362	65.9 [36.3–86.8]	93.0 [59.8–99.2]	0.683ϕ
Primary unit of analysis					
Per-artery [6, 23–25]	4	912	63.1 [46.3–77.2]	94.6 [61.7–99.5]	
Per-patient [34, 36]	2	202	70.3 [41.3–88.9]	96.1 [86.5–989]	< 0.001§
Per-artery and per-patient [20, 35, 37]	3	859	65.9 [36.3–86.8]	93.0 [59.8–99.2]	0.035¥

*Test of comparison between <16 and 16 slices

ϕTest of comparison between <16 and >16 slices

§Test of comparison between per-artery and per-patient

¥Test of comparison between per-artery and per-artery and per-patient

## Discussion

This systematic review and meta-analysis showed low sensitivity and moderate to good specificity for CTA (Table 5) in diagnosing BCVI as defined by DSA (Table 6). This is in line with previously published results on the diagnostic accuracy between CTA and DSA for low-channel CTA. However, contrary to our hypothesis and previous results by Paules et al, this study did not find that an increase of CTA channels beyond 16 slices showed higher diagnostic accuracy in detecting BCVI. A possible explanation for the observed low sensitivity is the absence of recent studies including both higher channel CTA (i.e., > 64 slices) and DSA as a reference test [38–40]. This could be attributed to the invasiveness of DSA and the possible complications associated with the technique, higher cost, and its availability in both equipment and expertise. Although one might expect a higher diagnostic accuracy with high-channel CTA, its intra- and interobserver reliability and the expected better yield of detection of BCVI are not established.

**Table 5:** The characteristics of computed tomography angiography (CTA) per study

CTA characteristics								
		Slices			Contrast			
Study	Scanner	No.	Thickness (mm)	Thickness interval (mm)	Type	Injection rate (mL/s)	Typical volume (mL)	Area scanned
	Toshiba Aquilion 64-channel computed tomographic scanners	64-neck 64-body	1.0	0.5	Optiray 320 (Guerbet LLC)	5	60-75 (neck), 120 (full body)	From the clavicles to the apex of the calvarium
	Toshiba Aquilion 32-channel computed tomography scanners	32	1	0.5	Optitray 320 (Guerbet LLC)	4	120	From the clavicles to the apex of the calvarium
	Toshiba Aquillion 64 detector scanner	64	1.0	0.5	Omnipaque 350 (GE Healthcare)	4	75–100	From the aortic arch to the circle of Willis
	General Electric (GE), Advantage Lightspeed 16-slice CT scanner	16	1.25	0.5	Omnipaque 350 (GE Healthcare)	4	85–125	From the aortic arch to the circle of Willis
	Philips Medical Systems, MX8000 IDT, Brilliance 16 Power, or Brilliance Big Bore	16-neck 16-body	NA	NA	Omnipaque 300 (GE Healthcare)	4	100	From the aortic arch to the circle of Willis
	Siemens Somatom Sensation-16 multidetector scanner	16	2	NA	Omnipaque300 (Amersham Health Inc)	4	80	From the aortic arch to the circle of Willis
	General Electric (GE) Advantage Lightspeed 16-channel CT scanner GE Medical Systems	16	1.25	0.55	Omnipaque 300 (Amersham Health Inc.)	3.5	125	From the aortic arch to the vertex of the head
	General Electric (GE) Lightspeed four-slice and eight-slice helical multidetector CT scanners and GE HighSpeed single-slice helical CT scanner.	1, 4, 8	1 to 3, mean 1.5	NA	NA	4	80–110	From the aortic arch to the circle of Willis
	Siemens Somatom 4 helical scanner	4	1	NA	NA	NA	125	Including both the aortic arch and the skull base
	General Electric(GE) Hilite scanner	1	NA	NA	Optiray320, (Mallinckrodt Pharmaceuticals)	2.5 for 20 s 1.75 for 60s	155	From the bottom of C3 to the sella turcica

*NA* not available, *CTA* computed tomography angiography

**Table 6:** The characteristics of digital subtraction angiography (DSA) per study

DSA characteristics			
		Contrast	
Study	Scanner	Type	Typical volume (mL)
Paulus et al [20]	Siemens AXIOM Artis biplane system	Optiray 320 (Guerbet LLC)	50–100
DiCocco et al [24]	Siemens AXIOM Artis biplane system	Optiray 320 (Guerbet LLC)	50–100
Goodwin et al (64 slices) [36]	Siemens Multistar Plus angiographic unit	Omnipaque 350 (GE Healthcare) and Visipaque 320 (GE Healthcare)	100–150
Goodwin et al (16 slices) [36]	Siemens Multistar Plus angiographic unit	Omnipaque 350 (GE Healthcare) and Visipaque 320 (GE Healthcare)	100–150
Sliker et al [25]	NA	NA	NA
Malhotra et al [37]	GE Advantix Biplane Angiography System	Omnipaque 300 (Amersham Health Inc.)	100–150
Eastman et al [35]	Siemens Artis BA biplane neuroangiographic unit	Omnipaque 300 (Amersham Health Inc.)	NA
Bub et al [23]	Philips biplane fluoroscopy tables with 12-inch image intensifier	NA	NA
Miller et al [6]	NA	NA	NA
Biffl et al [34]	NA	NA	NA

*NA* not available, *DSA* digital subtraction angiography

Due to its widespread availability, lower invasiveness, and cost-effectiveness, CTA is already widely used in clinical practice to detect BCVI. The complex hemodynamic nature of BCVI, the applied protocols, and guidelines have mainly focused to rule out any patients with a false negative finding for BCVI with CTA, thus eliminating the need for anticoagulation treatment. Although DSA is still presumed to be the golden standard, it is selectively and mostly used to confirm the presence of BCVI in either clinically suspected cerebrovascular injury with negative CTA or to better visualize a highly suspected BCVI on DSA. Therefore, the possibility and desirability of replacing DSA for CTA as the golden standard in diagnosing BCVI is still a subject of discussion.

The use of DSA to only confirm the presence of BCVI on CTA raises other concerns. Studies have shown that neurological symptoms in patients with BCVI might be delayed [12, 13]. Due to the low sensitivity of CTA shown in this study, a substantial number of patients with a false-negative outcome for BCVI are missed, which would leave them untreated. Hence, the pivot question seems to be how harmful undertreatment is in undiagnosed BCVI patients. Assuming that high-grade BCVIs are detected on both DSA and CTA imaging, patients with low-grade BCVI (such as intimal irregularity or intramural hematoma) would be most at risk for undertreatment. However, despite the available recommendations to treat this patient category either with aspirin or heparin for secondary prevention of thrombus formation [39, 41, 42], there is no established evidence that this has a beneficial effect in preventing cerebrovascular events.

This study has some important limitations. First, due to the heterogenic nature of the population in different studies and the difference in sample size, it is difficult to extrapolate the finding to the general trauma population. The use of different equipment with different settings and contrasts limits the applicability of the results to all kinds of equipment used in the field, especially when outdated CT scanners are increasingly being replaced by scanners with 256-detector rows, low-kVp imaging, multi-energy reconstruction, and all kinds of post-processing 3D reconstruction technology. Also, nontrivial settings such as the velocity of injection of the contrast might have influenced the diagnosis of BCVI. Future studies should therefore focus on more modern scanners and standardized protocols.

Second, the human factor is even more important in visualizing subtle changes in imaging. There is no evidence on how accurate radiologists or other specialists are in diagnosing (mild) BCVI in CTA. Subsequently, factors such as special interest, experience, exposure, and central referral might influence the accuracy to visualize even small and subtle changes in imaging [43]. This should be the focus of future studies to rule out the bias introduced by human inconsistency and establish minimum requirements in caregivers to establish a reliable and reproducible statement.

Third, there is a risk of selection bias when including only patients diagnosed with both CTA and DSA. It is possible that, due to time restrictions, patients with polytrauma suspected for BCVI were not included for both CTA and DSA screening. This could lead to an underrepresentation of patients with high-grade BCVI or even those with low-grade dissections that are only screened when severe and/or delayed neurological symptoms occur.

In conclusion, this systematic review and meta-analysis showed moderate to good specificity but low sensitivity of CTA in diagnosing BCVI compared to DSA. Furthermore, CTA with higher channels (16-64) did not increase the diagnostic accuracy of CTA compared to lower channels (<16). This might lead to a risk of undertreatment of BCVI in false-negative cases, especially in those with low-grade injuries. It is unclear whether this is associated with an increased risk of cerebrovascular events. Future studies should focus on a. the diagnostic accuracy of nowadays widely available 256-channel CTA, the inter- and intra-observer reliability, and on the harmfulness of undertreatment of BCVI patients.

## Abbreviations

BCVI: Blunt cerebrovascular injury
CTA: Computed tomography angiography
DSA: Digital subtraction angiography

## Declarations

### Guarantor

The scientific guarantor of this publication is W. B. M. Slooff.

### Statistics and biometry

dr. Paco M. J. Welsing, associate Professor of Clinical Epidemiology at University Medical Center Utrecht kindly provided statistical advice for this manuscript.

### Methodology

- retrospective
- diagnostic or prognostic study
